# Caries of the Petrous Portion of the Temporal Bone

**Published:** 1851-07

**Authors:** 


### ARTICLE XXVII.

### *Caries of the Petrous Portion of the Temporal Bone.*

Mr. Willing, of Hampstead, detailed at some length the case of a child, eleven months old, in whom there occurred caries of the petrous portion of the right temporal, implicating the spinous process of the sphenoid, and part of the basilar portion of the occipital, with complete disorganization of the vessels and nerves passing through their foramina. In addition to the signs of general ill health, the child had long been subject to a discharge from the right ear, and some months before death the left side of the face was palsied, the loss of power being more evident a week prior to disease. Pain was evidently felt on pressure on the mastoid. All these symptoms increased, the discharge became profuse, bloody, and very offensive, the pain on pressure more severe, and the parts yielded to the touch.

The child was sensible to within twelve hours of her decease, but died convulsed. The post-mortem appearances were those of congestion and effusion into the brain, a small abscess at the posterior part of the right middle lobe, while the bones were as above described.—*London Lancet.*

